# Association between endoscopic cyclophotocoagulation and vitreous prolapse in trabeculectomy: a case report

**DOI:** 10.1186/s12886-022-02363-5

**Published:** 2022-03-26

**Authors:** Vikki W. K. Ng, Jeffrey C. W. Chan, Kenneth K. W. Li

**Affiliations:** Department of Ophthalmology, United Christian Hospital Hong Kong SAR, 130 Hip Wo Street, Kwun Tong, Kowloon, Hong Kong SAR, Hongkong

**Keywords:** Endoscopic cyclophotocoagulation, Zonular damage, Trabeculectomy, Vitreous prolapse, Case report

## Abstract

**Background:**

To propose that possible alteration or damage to the ciliary zonules during uncomplicated endoscopic cyclophotocoagulation (ECP) can cause complications in subsequent filtration surgery.

**Case presentation:**

We present two cases with uncomplicated primary combined phacoemulsification and ECP that underwent subsequent trabeculectomy. Both cases were complicated with vitreous prolapse during the trabeculectomy procedure. We review the anatomy of the ciliary zonules and their spatial relationship with the ciliary processes targeted during ECP and propose an association between ECP, zonular damage and complications in subsequent trabeculectomy such as vitreous prolapse.

**Conclusions:**

Damage to ciliary zonules during ECP may not manifest until subsequent glaucoma filtration surgery. In patients who received prior ECP, we may consider taking preventative measures to avoid associated complications such as vitreous prolapse. Patients with known risk factors for weak zonules may consider choosing alternative intraocular pressure-lowering means.

## Background

Vitreous prolapse is an uncommon complication seen in less than 0.5% of glaucoma filtration surgery [1]. Possible risk factors include aphakia, a subluxed or dislocated lens or eyes with underlying zonular weakness. Zonular weakness can be secondary to ocular trauma, prior ocular surgery, high myopia, ocular diseases such as uveitis and pseudoexfoliation syndrome, as well as systemic connective tissue diseases such as Marfan syndrome.

We observed two eyes over a period of 10 years with the complication of vitreous prolapse during glaucoma filtration surgery, both of which received prior endoscopic cyclophotocoagulation (ECP). During ECP, the ciliary processes are targeted for treatment with a diode laser. The zonules are in close proximity to the ciliary processes and some even originate from its lateral walls. Yet clinically significant damage to zonules during uncomplicated ECP leading to subsequent complications is seldom suggested.

We propose that ECP could potentially weaken or damage the zonular integrity which may be a precipitating factor for vitreous prolapse in glaucoma filtration surgery. This knowledge may alter our future management. Procedures other than ECP can be considered in patients with underlying risk factors to prevent further exacerbation of zonular weakness. In eyes with prior ECP, preventative measures against vitreous prolapse should be taken if glaucoma filtration surgery is required.

## Case presentation

### Case 1

A 75-year-old man with bilateral advanced primary open angle glaucoma (POAG) received a combined phacoemulsification and ECP surgery in his left eye in 2014. The eye had + 2.5 dioptres (D) of hyperopia (axial length 22.15 mm) and he had no other ocular or systemic diseases or trauma which would predispose him to zonulopathy. There was also no pre-existing phacodonesis or vitreous noted in the anterior chamber.

The surgery was uneventful: phacoemulsification was completed via a temporal 2.2 mm clear-corneal incision. Viscoelastic was then injected to open up the sulcus space and three-hundred degrees of ECP was performed from 4 to 2 o’clock. The power was set at 0.3 W and 94 shots were fired at approximately 0.5 to 2 s per shot. Post-operatively, the IOP was reduced without hypotony and well controlled until 2-years post-ECP.

This eye then underwent a trabeculectomy. A fornix-based conjunctival flap was created in the superonasal quadrant, a partial-thickness scleral flap was raised and mitomycin-C (0.4 mg/ml) soaked pledgets were placed subconjunctivally for 3 min. After irrigation with 100 ml of balanced salt solution (BSS), a paracentesis at 9 o’clock was created. A sclerotomy was made with a Kelly punch and a peripheral iridectomy was created with Vannas scissors. While closing the scleral flap with 10/0 nylon sutures, vitreous prolapse through the sclerotomy was seen underneath the scleral flap. Scleral flap closure was completed, and thorough manual Weck-cel vitrectomy was performed with Westcott scissors. The conjunctiva was closed with interrupted 8/0 vicryl sutures. Post-operatively, there was no vitreous found in the anterior chamber or around the sclerotomy site and the intraocular pressure (IOP) was in the mid-teens. He remained drop-free for 4 months, and has now maintained a pressure of around mid-teens on anti-glaucomatous drops without the need for further glaucoma surgery thus far.

### Case 2

An otherwise healthy 58-year-old man with bilateral POAG underwent combined phacoemulsification and ECP in his right eye in 2013. As above, he had no predisposing risk factors for or pre-existing zonulopathy. He was mildly myopic (-1.75D, axial length 24.65 mm). The surgery was uncomplicated with the same surgical steps as described in Case 1. 97 shots of ECP at 0.35 W was applied from 4 to 2 o’clock. Similarly, initial IOP control was good with no hypotony but gradually began to rise. In 2015, he underwent a non-penetrating trabecular surgery which was converted into a trabeculectomy due to macro-perforation.

**Fig. 1:**
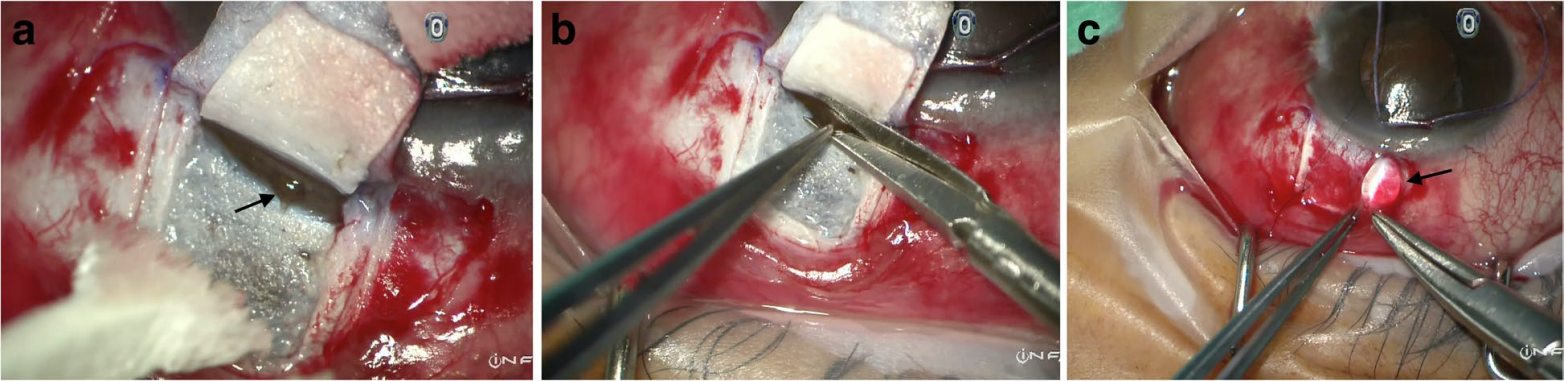
**A** Macro-perforation with iris prolapse (black arrow) during peeling of the inner wall of Schlemm’s canal and juxtacanalicular meshwork. **B** Surgical iridectomy performed over the prolapsed iris at the perforation site. **C** Vitreous prolapse (black arrow) during closure of the scleral flap

The conjunctiva was opened with a fornix-based approach and subconjunctival Mitomycin-C (0.4 mg/ml) was applied in the same fashion after the partial-thickness superficial and deep scleral flaps were created. During the peeling of the inner wall of the Schlemm’s canal and juxtacanalicular meshwork, a macro-perforation occurred (Fig. 1A). Viscoelastic and carbachol (Miostat) were used to maintain the anterior chamber depth and constrict the pupil respectively. A peripheral iridectomy was created through the perforation site (Fig. 1B) and the deep scleral flap was excised. The superficial scleral flap was then closed with 10/0 nylon sutures and vitreous prolapse was again noted at this stage (Fig. 1C). It was managed similarly as in the previous case by manual Weck-cel vitrectomy. Post-operatively, the IOP was well-controlled with no vitreous seen in the anterior chamber or blocking the drainage pathway. He remained drop-free for 4 months, and now has intraocular pressure controlled around mid-teens on anti-glaucomatous drops without further glaucoma surgery required thus far.

## Discussion and conclusions

ECP was first reported in literature for the treatment of uncontrolled neovascular glaucoma by Uram in 1992 [2]. It is a surgically non-demanding procedure shown to be both safe and effective and is commonly performed in early- to mid-stage POAG [3–5]. Even in cases with refractory glaucoma where filtration surgeries have failed, ECP is safe [6] and can reduce IOPs up to 34% at 1-year post-procedure follow up [7]. In one of our previous publications, we found that combined phacoemulsification and ECP when compared to combined phacoemulsification and trabeculectomy had lower complete success rates, but similar qualified success rates. (73.5% vs 74.4%) [8]. Complications of ECP that were reported by the ECP Collaborative Study Group included IOP spikes (14.5%), hyphema (3.8%), choroidal detachment (0.36%) and cystoid macular edema (0.7%). In their study, other severe complications such as retinal detachment (0.2%), choroidal haemorrhage (0.09%) and hypotony (0.12%) were only found in eyes with neovascular glaucoma [9].

This procedure is performed with an intraocular endoscope paired to a diode laser and the ciliary processes are approached ab-interno. The visible portions of the ciliary processes are then treated with continuous laser applied in a painting motion until whitening and shrinkage of the tissue is observed. As a result, IOP can be reduced by diminishing aqueous production [10]. Depending on the target IOP, it can be performed through a one-site corneal incision to cover 180 to 300 degrees of ciliary processes or two-site corneal incisions to treat all ciliary processes (360 degrees). It has been shown that 360-degree treatment resulted in significantly lower IOP without increased incidence of complications [11].

Possible alteration to zonular anatomy secondary to ECP has been suggested in a previous study by Sheybani A et al [12]. It looked at the effect of ECP on refractive outcomes in cataract surgeries performed on open angle glaucoma patients. Their results showed a significantly larger difference between predicted and actual refractive outcomes in eyes that underwent combined phacoemulsification and ECP than those with phacoemulsification alone. Eyes that underwent the combined procedure demonstrated a myopic shift (-0.169D to -0.312D in combined phacoemulsification and ECP group vs + 0.029D to -0.110D in phacoemulsification alone group [*p* < 0.05]). The authors hypothesized that as the lens zonules are attached to the ciliary body, an alteration in the ciliary body position after ECP induces a change in the position of the capsular bag-intraocular lens complex and therefore the effective lens position and refractive outcome. Another study by Manoharan N et al [13] showed no significant difference in number of refractive surprises between combined phacoemulsification and ECP and phacoemulsification alone. However, they defined “significant” as a difference of greater than 1D. Therefore, their results do not directly contradict those of Sheybani A et al. and any statistically significant refractive surprise of less than 1D is unknown. In both of our cases, a myopic shift was noted—case 1 had a target refraction of spherical equivalent (SE) -0.51D but outcome at 1 month was -2D; case 2 had a target refraction of SE -0.53D and outcome at 1 month was -1.38D. These outcomes corroborate the findings of Sheybani A et al. but whether this shift directly translates to zonular damage would require further investigation.

**Fig. 2:**
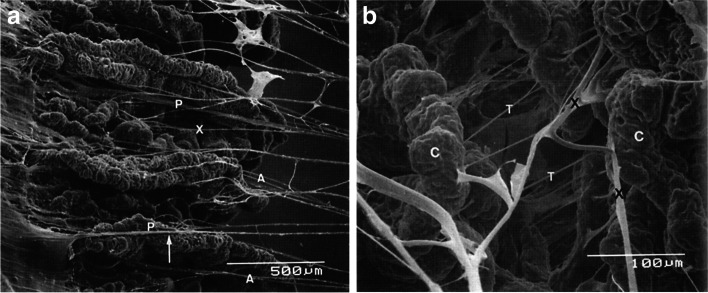
**A** Scanning electron micrograph of pars plicata of the ciliary body. Anterior zonular fibres (A) are attached to the lateral walls of the ciliary processes and emerge at their anterior endings. Fibres originating at the ciliary valleys are observed (X). × 49. (Reused with permission from Canal M [15], Copyright Karger Publishers). **B** Scanning electron micrograph. At a higher magnification, transversal fibres (T) can be seen between adjacent ciliary processes (C). Fibres originating at the lateral walls of the ciliary processes belonging to the anterior zonular layer are seen (X). × 240. (Reused with permission from Canal M [15], Copyright Karger Publishers)

The zonular apparatus of the lens consists of anterior and posterior radial fibres connecting the lens to the ciliary body. They insert into the anterior and posterior lens capsules respectively. Over the years there have been conflicting opinions on the origins and insertions of the zonular fibres. It is largely accepted that the primary origin of these fibres is the pars plana [14]. Electron microscopy performed by M. Canals et al [15] showed that the zonular fibres traverse through the ciliary valleys before inserting into the lens capsule—the anterior fibres in particular are in close proximity to the lateral walls of the ciliary processes during their journey. Furthermore, although most fibres originate from the pars plana, some of the anterior fibres originate from the ciliary valleys or the lateral walls of the ciliary processes themselves (Fig. 2A & 2B). Therefore, anatomically, alteration of or damage to the zonular fibres during ECP is certainly possible. The resultant weakening of zonular integrity—although may not cause immediate complications—may manifest when additional stress to the zonules is applied. An example of when this may occur is during glaucoma filtration surgery, then complications such as vitreous loss or zonulysis may arise.

A few other studies have also touched upon zonular damage during ECP. Lindfield D et al [16] noticed vitreous in the anterior chamber in one patient on postoperative day one after combined phacoemulsification and ECP. The authors postulated that it was caused by a small area of zonular dehiscence during surgery. This could have been induced by either the phacoemulsification or the ECP. Morales J et al [17] reported another case of combined phacoemulsification and ECP where intraoperative zonular dialysis was noted during the procedure. However, in these reports, the complications were noted during or immediately after the primary procedure and neither elaborated on the mechanism of zonular injury or reported any complications in subsequent procedures.

In our two cases reported above, the primary ECP procedure was seemingly uncomplicated intra- and post-operatively, with no signs of zonular damage such as vitreous loss, zonulysis or intraocular lens decentration. It was not until the second-stage glaucoma filtration surgeries that vitreous prolapse through the sclerotomy upon creation of a surgical iridectomy was noted. Incidence of vitreous prolapse as a complication of trabeculectomy is seldom cited in literature and is considered to be rare. In our center, 242 trabeculectomies were done from 2010–2020 and 44 had prior ECP. Only the 2 cases illustrated above were complicated with vitreous prolapse making the overall incidence of vitreous prolapse during trabeculectomy with or without prior ECP 0.8% (2/242) and in those with prior ECP 4.5% (2/44). The route of prolapse begins when vitreous from the vitreous cavity enters the posterior chamber. Under normal anatomical circumstances, this communication is blocked by the zonules.

Neither patient had pre-existing risk factors for zonulopathy. We propose that the ECP was the culprit behind the zonular damage precipitating vitreous prolapse. During filtration surgery, there is a sudden drop in pressure when the sclerotomy is created. This generates a large pressure gradient, inviting the vitreous to prolapse through the weakened zonules. The surgical iridectomy then creates a communication between the posterior and anterior chambers, allowing the vitreous to enter the anterior chamber and prolapse through the sclerotomy.

Another possible cause of vitreous prolapse in trabeculectomy is surgical damage to the zonules or ciliary body during sclerotomy or surgical iridectomy. A sclerotomy fashioned too posteriorly can cause direct damage to the zonules or ciliary body. We believe this to be an unlikely cause in the cases illustrated above as no direct damage was noted intra-operatively and post-operative gonioscopy assessment did not reveal abnormally posterior sclerotomies.

**Fig. 3:**
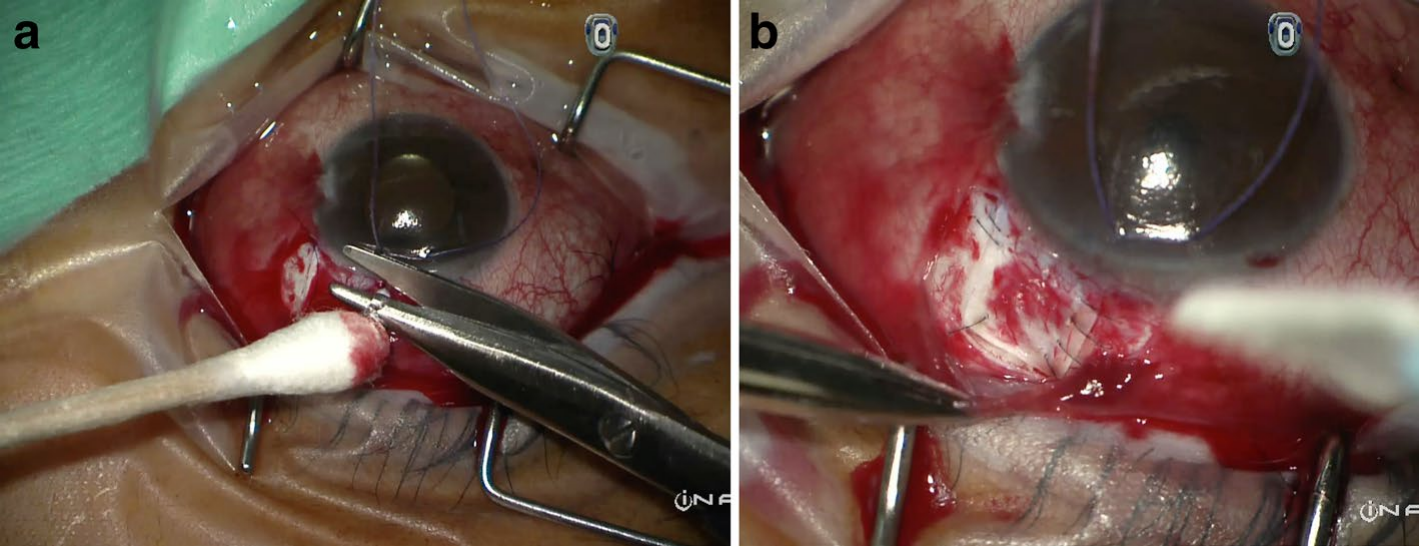
**A** Manual vitrectomy by cotton tip applicator and Weck-cel to remove all vitreous at the edges of the scleral flap. **B** The scleral flap was tied down tight with multiple stitches to avoid postoperative hypotony and vitreous blockage of the sclerotomy

Given the low incidence of vitreous loss during glaucoma filtration surgery, there is no consensus on its management approach. In our cases, after vitreous prolapse was noted, we maintained the anterior chamber with a cohesive viscoelastic. We then simply removed only the prolapsed vitreous at the edges of the scleral flap by manual Weck-cel vitrectomy (Fig. 3A). No anterior vitrectomy was performed. We then closed the scleral flap with a few more stitches and at a higher tension than usual (Fig. 3B) in order to minimize the aqueous outflow to avoid too steep of a pressure gradient which may lead to vitreous blockage of the sclerotomy. In both cases—with a well formed anterior chamber and the IOP at mid-teens—the vitreous retracted back into the vitreous cavity. IOP was then well-maintained until their latest follow up more than 4 years later with no further glaucoma surgeries required. In our opinion, when vitreous prolapse does occur the extent is minimal and can be managed without anterior vitrectomy. Early fundal examination was performed and is recommended to look for possible complications of vitreous traction secondary to vitreous prolapse such as retinal breaks.

Vitreous loss during surgery can potentially lead to multiple sight-threatening complications such as suprachoroidal haemorrhage, retinal detachment, cystoid macular edema and endophthalmitis. It also increases the risk of failure in glaucoma filtration surgery due to vitreous blockage of the sclerotomy. Therefore, methods to prevent this complication have a significant impact on the visual outcome of our patients. We have proposed that damage or weakening of the zonules can occur even in apparently uncomplicated ECP. Caution is advised when considering ECP for patients with high baseline IOP where the potential need for subsequent glaucoma filtration surgery is significant. In patients who have had prior ECP and require glaucoma filtration surgery, the anticipation of vitreous prolapse can prompt surgeons to adopt preventative measures so that sight-threatening complications can be avoided and surgical outcomes improved. For example, stabilising the anterior chamber with the use of an anterior chamber maintainer or injection of viscoelastic prior to sclerotomy can be considered. This can dampen the sudden drop in intraocular pressure and therefore minimize the pressure gradient that encourages the vitreous to prolapse. Alternatively, other surgical procedures such as glaucoma drainage device implantation rather than trabeculectomy can be considered in patients with prior ECP.

## Data Availability

The datasets used and analysed during the current study available from the corresponding author on reasonable request.

## Abbreviations

ECP: Endoscopic Cyclophotocoagulation
POAG: Primary Open Angle Glaucoma
D: Dioptres
BSS: Balanced Salt Solution
IOP: Intraocular Pressure
SE: Spherical Equivalent
